# Potential mechanism of Taohong Siwu Decoction in preventing and treating postoperative delirium in intertrochanteric fracture patients based on retrospective analysis and network pharmacology

**DOI:** 10.1186/s13018-024-04854-1

**Published:** 2024-06-21

**Authors:** Zhihong Ding, Zhiyong Yu, Zhibo Sun, Xinghui Liu, Rong Chen

**Affiliations:** 1 Department of traumatic orthopedics, Xiangyang Hospital of Traditional Chinese Medicine [Xiangyang Institute of Traditional Chinese Medicine], Xiangyang, 441000 Hubei China; 2 School of Basic Medical Sciences, Hubei University of Arts and Science, No. 296 Longzhong Road, Xiangcheng District, Xiangyang, 441000 Hubei China

**Keywords:** Taohong Siwu Decoction, Postoperative delirium, Intertrochanteric fractures, Inflammation

## Abstract

**Objective:**

Elderly patients with hip fractures are at a greater risk of developing postoperative delirium (POD), which significantly impacts their recovery and overall quality of life. Neuroinflammation is a pathogenic mechanism of POD. Taohong Siwu Decoction (THSWD), known for its ability to promote blood circulation and remove blood stasis, can effectively reduce inflammation in the nervous system. Therefore, the objective of this article is to provide a comprehensive summary of the clinical efficacy of THSWD in the prevention of POD. Additionally, it aims to investigate the underlying mechanism of THSWD in the prevention and treatment of POD using network pharmacology and molecular docking.

**Methods:**

We conducted a retrospective analysis of patients with intertrochanteric fractures between January 2016 and October 2021. The patients were divided into two groups: the control and THSWD group. We performed a comparative analysis of hemoglobin (HB), albumin (ALB), C-reactive protein (CRP), blood urea nitrogen (BUN), and the blood urea nitrogen to creatinine ratio (BCR) on two different time points: the day before surgery (D0) and the third day after surgery (D3). Furthermore, we examined the incidence and duration of delirium, as well as the Harris Hip Score (HHS) at 3 months and 12 months post-surgery. Network pharmacology was employed to identify the primary targets and mechanisms of THSWD in the management of delirium. Molecular docking was employed to confirm the interaction between active ingredients and COX-2. Inflammatory cytokines, including cyclooxygenase-2 (COX-2), interleukin-1β (IL-1β), interleukin-6 (IL-6), and tumor necrosis factor- (TNF-α), were measured using the enzyme-linked immunosorbent assay (ELISA). The cognitive status of the patients was assessed using the Mini-Mental State Examination (MMSE) scoring system.

**Results:**

Regardless of whether it is in D0 or D3, THSWD treatment can increase HB levels while decreasing BCR. In D3, the THSWD group demonstrated a significant reduction in the expression of CRP and BUN when compared to the control group. However, there were no significant differences in ABL levels, surgery duration, and blood loss between the two groups. Additionally, THSWD treatment requires fewer blood transfusions and can reduce the incidence and duration of POD. The results of the logistic analysis suggest that both CRP levels and BCR independently contribute to the risk of POD. Network pharmacology analysis indicates that THSWD has the potential to prevent and treat POD possibly through inflammatory pathways such as IL-17 signaling pathways and NF-kappa B signaling pathways. Molecular docking validated the interaction between the active ingredient of THSWD and COX-2. Furthermore, THSWD treatment can reduce the levels of COX-2, IL-1β, IL-6, TNF-α, BUN and CRP in the blood of patients with POD, increase HB levels, and enhance MMSE scores. The expression of COX-2 is positively associated with other inflammatory markers (IL-1β, IL-6, TNF-α, and CRP), and inversely associated with MMSE.

**Conclusion:**

THSWD has been found to have a preventive and therapeutic effect on POD in intertrochanteric fracture patients possibly through inflammatory pathways. This effect may be attributed to its ability to increase hemoglobin levels and reduce the levels of certain detrimental factors, such as blood urea nitrogen and inflammatory factors.

## Introduction

Hip fractures are the primary cause of hospitalization among elderly patients. Comprehensive perioperative care, involving multidisciplinary collaboration, early surgical intervention, pain management, fluid balance therapy, and delirium prevention, can decrease mortality rates and prevent complications [1]. Intertrochanteric fractures are common in the hip, and the use of intramedullary nails for fixation has been widely acknowledged by researchers [2, 3]. However, elderly patients with hip fractures may undergo substantial muscle mass loss and a decline in activities of daily living (ADL) one year post-surgery [4]. Additionally, Delirium is a commonly observed neuropsychiatric syndrome that abruptly occurs in elderly patients with hip fractures. It is characterized by attention deficits, disturbances in consciousness, and cognitive impairments [5]. Postoperative delirium (POD) typically manifests 2 to 5 days after hip fracture surgery [6], with an incidence rate ranging from 16.9–70% [7–9]. In elderly patients with hip fractures, POD is associated with an increased risk of medical comorbidity, longer hospital stays, higher postdischarge mortality rates, and greater medical burden [10–12]. However, the underlying causes of delirium following hip fracture surgery in the elderly remain poorly understood, and there are currently limited treatment options available for POD. Low-dose dexmedetomidine and multimodal analgesia after surgery may be effective in managing POD, while the use of antipsychotics may have more risks than benefits [13]. Moreover, the occurrence of POD is influenced by a complex interplay of various risk factors, including traumatic stress, pain, advanced age (> 70 years), previous diagnosis of dementia or cognitive impairment, multiple comorbidities, low albumin levels, anemia, hypoxemia, preoperative waiting time, and duration of anesthesia [14–16]. Despite various proposed measures, such as Orthogeriatric comprehensive care, the availability of pharmacological interventions to decrease the occurrence of POD remains limited [6, 13]. While Traditional Chinese Medicine (TCM) is widely acknowledged for its efficacy in treating numerous ailments, there is a scarcity of literature reports investigating its potential in preventing POD.

According to the theory of TCM, delirium is classified as a form of “madness”, with blood stasis being its primary pathogenic factor. Taohong Siwu Decoction (THSWD), composed of six herbs including Angelicae Sinensis Radix (Danggui), Rehmanniae Radix Praeparata (Shudihuang), Paeoniae Radix Alba (Baishao), Chuanxiong Rhizoma (Chuanxiong), Persicae Semen (Taoren), and Carthami Flos (Honghua), has been shown to improve blood circulation and alleviate blood stasis [17]. Furthermore, mounting evidence indicates that THSWD exhibits various pharmacological activities in the treatment of neurological disorders, encompassing antioxidant, anti-apoptotic, and anti-inflammatory effects [18–20]. Neuroinflammation is widely recognized as a pathophysiological mechanism underlying POD in elderly patients with hip fractures. Surgical trauma triggers an intracellular inflammatory response, resulting in the release of inflammatory mediators, including TNF-α, into the brain, ultimately culminating in the development of POD [21].

Therefore, the aim of the present study was to retrospectively analyze the potential improvement in the prognosis of patients with POD after intertrochanteric fracture (a type of hip fracture) surgery by using THSWD. Furthermore, network pharmacology was employed to identify the key targets and mechanisms through which THSWD prevents POD. This study offers novel insights into the preventive effects of THSWD on POD.

## Materials and methods

### Inclusion and exclusion criteria

**Fig. 1:**
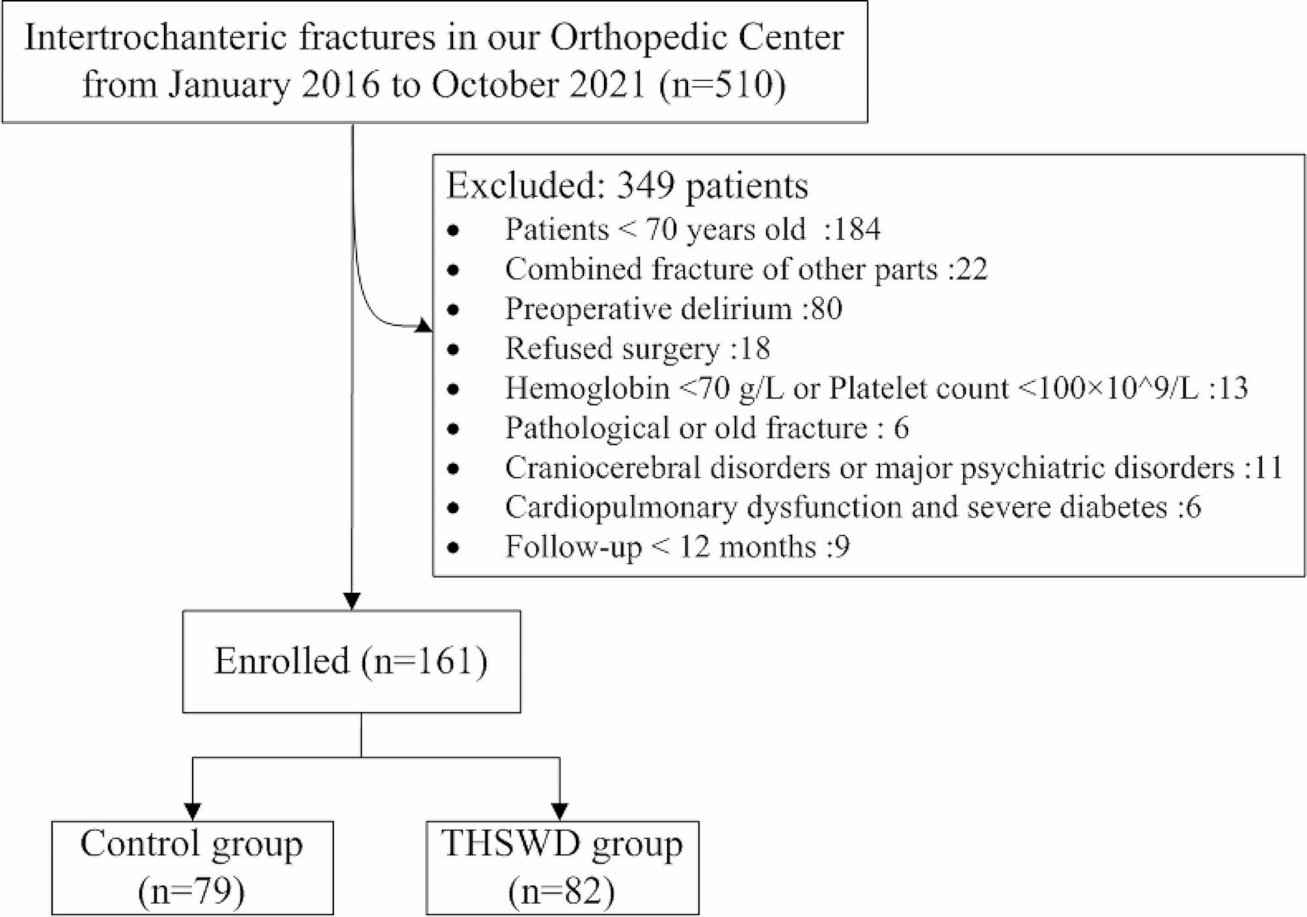
Study flow chart

The clinical data of patients diagnosed with femoral intertrochanteric fracture in the Department of Orthopedics at Xiangyang Hospital of Traditional Chinese Medicine from January 2016 to October 2021 were retrospectively collected (Fig. 1). The inclusion criteria were as follows: (1) X-ray and CT examination confirmed an isolated intertrochanteric fracture. (2) Patients aged 70 or above who received proximal femoral nail anti rotation (PFNA) treatment with a minimum of 1-year follow-up. (3) Hemoglobin concentration was not less than 70 g/L, and platelet count was not less than 100 × 10^^9^/L. (4) Patients were willing to sign informed consent forms and cooperate with medical staff to complete relevant diagnosis and treatment protocols. The exclusion criteria included: (1) Preoperative delirium. (2) Patients with pathological, open or old fractures. (3) Patients had acute or chronic craniocerebral disorders, such as acute cerebral hemorrhage, coma, premorbid dementia, previous cerebral infarction, Parkinson’s and Alzheimer’s disease, and major psychiatric disorders such as schizophrenia, bipolar disorder, and acute major depression. (4) Patients with cardiopulmonary dysfunction and severe diabetes. (5) Patients who refused surgery. Since this study is retrospective and involves the analysis of blood-related indicators in patients without causing harm, the Ethics Committee of Xiangyang Hospital of Traditional Chinese Medicine deems an ethical application unnecessary. Delirium diagnosis relies on the Confusion Assessment Method (CAM) [22] for extracting characteristic words from the patient’s medical records. 161 enrolled patients were retrospectively divided into the Control group and the THSWD group depending on whether they received THSWD treatment after admission.

### Patient management

Both groups of patients received routine orthopedic care, analgesia, sedation, correction of electrolyte abnormalities, fluid therapy, and postoperative infection prevention therapy. Patients with underlying diseases such as hypertension and diabetes may require adjustments for tolerable surgery with the assistance of relevant departments. All patients underwent PFNA surgery under spinal anesthesia.

### Data collection

Two authors independently reviewed all enrolled patients using the eligibility criteria, and any discrepancies were resolved by rechecking the data.

1) General information included age, sex, daily living (ADL) score, body mass index (BMI), hypertension, diabetes, chronic obstructive pulmonary disease (COPD), and time from admission to surgery.

2) The data collected on the day before surgery (D0) and on the third day after surgery (D3) included measurements of hemoglobin (HB), serum albumin (ALB), C-reactive protein (CRP), blood urea nitrogen (BUN), and blood creatinine (CREA). Prior to calculating the urea nitrogen to creatinine ratio (BCR), it is essential to standardize the units of measurement for both urea nitrogen and creatinine.

3) Gather data including surgical duration, blood loss, transfusion volume, delirium onset and duration after surgery, and Harris Hip Score (HHS) at 3 and 12 months postoperatively.

### Network pharmacological analysis

We searched for the chemical compositions of THSWD in the TCMSP database (https://old.tcmsp-e.com/tcmsp.php) and screened them based on an oral bioavailability (OB) of 30% and a drug-likeness (DL) of 0.18. We obtained the canonical SMILES of the chemical composition from the PubChem database (https://pubchem.ncbi.nlm.nih.gov/). Next, we used the SwissADME platform (http://www.swissadme.ch/) to filter the compounds based on a “high” GI absorption score and a drug-likeness of at least 2 “Yes”. Finally, we entered the canonical SMILES of the chemical composition into the Swiss TargetPrediction database(http://www.swisstargetprediction.ch/)to predict its targets. The genetic information was verified in the UniProt database, and genes from non-human species were eliminated. Additionally, disease targets associated with “delirium” were obtained from the Gene Cards database (https://www.genecards.org/) and DisGeNET database (https://www.disgenet.org/home/). The protein-protein interaction (PPI) analysis network for common targets of drugs and diseases was constructed using the STRING database(https://string-db.org/), with Homo sapiens selected as the background organism and default settings for other parameters. Network visualization was performed using Cytoscape 3.8.2 software. Furthermore, the DAVID database (https://david.ncifcrf.gov/tools.jsp)was utilized for Gene Ontology (GO) function enrichment and Kyoto Encyclopedia of Genes and Genomes (KEGG) pathway analysis.

### Molecular docking

The 2D chemical structure of the active ingredient is obtained from PubChem Compound and converted into a 3D structure using ChemBio3D (14.0.0.117), saved in MOL2 format. Only one protein target (COX-2) was investigated. Receptors and ligands were converted from their native format to pdbqt format using AutoDockTools 1.5.6. The structure was optimized by removing water molecules and adding hydrogen atoms. Subsequently, molecular docking studies were performed with Autodock. All docking operation options were set to default values according to the genetic algorithm. The docking results with the highest scores were visualized using PyMoL.

### Measurement of COX-2, IL-1β, IL-6, and TNF-α

Blood samples were collected from patients when they developed delirium and on the 5th day after delirium onset. The samples were then immediately centrifuged at 3000 rpm for 15 min, and the resulting supernatant was used for subsequent analysis. The concentrations of cyclooxygenase-2 (COX-2), interleukin-1β (IL-1β), interleukin-6 (IL-6), and tumor necrosis factor-α (TNF-α) in the supernatants were measured using commercial enzyme-linked immunosorbent assay (ELISA) kits(Solarbio, China), following the instructions provided by the manufacturer.

### Mini-mental state examination (MMSE)

The cognitive status of the patients was assessed by two physicians using the MMSE scoring system immediately after the onset of delirium and again on the 5th day after the onset. The MMSE has a maximum score of 30 points, with scores between 28 and 30 indicating normal cognition, 24 to 27 indicating mild cognitive impairment, 19 to 23 indicating moderate cognitive impairment, and 0 to 18 indicating severe cognitive impairment [23].

### Statistical analysis

The data were analyzed using SPSS version 25.0 (IBM Corporation, USA). The data were presented as mean ± SD. Measurement data are expressed as percentages, and rates were compared using the χ2 test. Inter-group comparisons were conducted using the t-test, while comparisons at different time points were analyzed using repeated measures analysis of variance. The binary logistic regression analysis method was used to analyze the relationship between several factors and the POD. The factors included in the analysis were age, ADL scores, BMI, duration from admission to surgery, surgical duration, blood loss, and transfusion volume, HB, ALB, BUN, CRP, and BCR. The analysis involved calculating hazard ratios and their corresponding confidence intervals. Differences were considered significant if the *P*-value was less than 0.05.

## Results

### General data

Overall, a total of 161 patients who fulfilled the eligibility criteria were enrolled in the study. This comprised of 82 patients in the THSWD group and 79 patients in the Control group (Fig. 1). Based on the statistical analysis, there were no significant differences in gender, age, ADL scores, BMI, underlying diseases, laboratory parameters, duration from admission to surgery, and AO classification of fractures between the Control group and the THSWD group (Table 1).

**Table 1:** Comparison of general demographics (mean ± SD; *n*, %)

	Control group (*n* = 79)	THSWD group (*n* = 82)	t/χ2	*P*
Gender			0.498	0.618
Male	25(31.65)	29(35.37)		
Female	54(68.35)	53(64.63)		
Age(years)	81.54 ± 5.28	81.41 ± 5.39	0.154	0.878
ADL scores	35.96 ± 2.37	36.40 ± 3.09	1.018	0.310
BMI(kg/cm^2^)	22.00 ± 1.82	22.48 ± 1.53	1.812	0.072
Underlying disease				
Hypertension	50(63.29)	51(62.20)	0.143	0.886
Diabetes	16(20.25)	19(23.17)	0.447	0.655
COPD	5(6.33)	7(8.54)	0.531	0.595
On admission				
Hemoglobin(g/L)	99.77 ± 8.93	99.56 ± 9.05	0.149	0.882
ALB(g/L)	35.74 ± 2.01	35.35 ± 2.19	1.202	0.231
BUN (mg/dl)	19.82 ± 1.55	19.60 ± 1.87	0.847	0.399
BCR	22.98 ± 2.35	23.59 ± 2.55	1.557	0.122
CRP(mg/L)	23.19 ± 12.40	22.90 ± 11.80	0.024	0.877
From admission to surgery(days)	4.07 ± 1.36	4.14 ± 1.62	0.78	0.781
AO classification of fractures			0.059	0.953
A1	28(35.44)	29(35.37)		
A2	39(49.37)	40(48.78)		
A3	12(15.19)	13(15.85)		

### THSWD increased hemoglobin levels and reduced levels of urea nitrogen and C-reactive protein

**Fig. 2:**
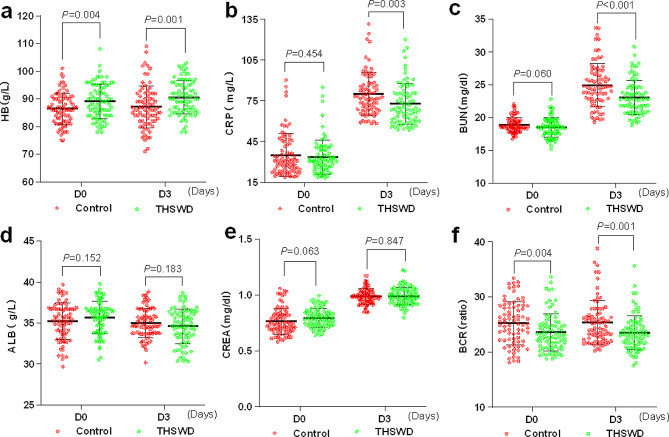
THSWD increased hemoglobin levels and reduced levels of urea nitrogen and C-reactive protein. **a-e** Expression levels of hemoglobin (HB), C-reactive protein (CRP), blood urea nitrogen (BUN), serum albumin (ALB), and blood creatinine (CREA) in the blood were measured on the day before surgery (D0) and the third day after surgery (D3). **f** The blood urea nitrogen to creatinine ratio (BCR)

Figure 2a demonstrates that the THSWD group had higher HB levels on both D0 and D3 compared to the Control group. Furthermore, there were no significant differences in CRP and BUN expression between the two groups on D0. However, on D3, the THSWD group exhibited a significant decrease in comparison to the Control group (Fig. 2b and c). Nevertheless, there were no statistically significant differences in ABL and CREA levels between the two groups on either D0 or D3 (Fig. 2d and e). Additionally, Fig. 2f illustrates a lower BCR in the THSWD group.

### THSWD had the potential to decrease the occurrence of POD and shorten its duration, as well as investigate the risk factors associated with POD

**Fig. 3:**
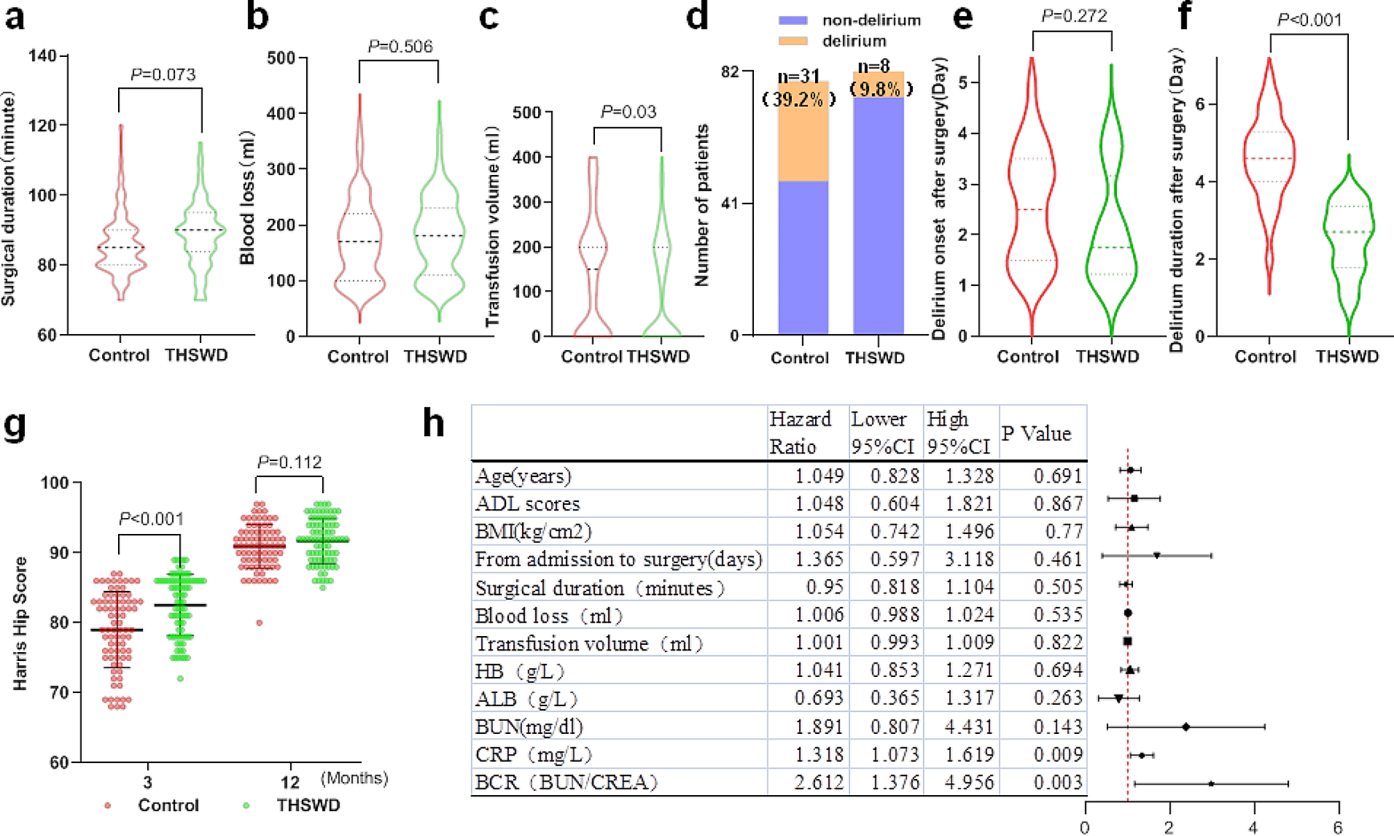
THSWD could lower the likelihood of POD and shorten its duration. **a** Surgical duration **b** Blood loss **c** Transfusion volume **d** Postoperative delirium incidence rate **e** Delirium onset duration after surgery **f** Delirium duration after surgery **g** Harris Hip Score (HHS) at 3 and 12 months after surgery. h The forest plot illustrates the hazard ratios of different factors along with their corresponding confidence intervals

Figure 3a and b showed no significant differences in the duration of surgery and blood loss between the two groups. In contrast, the THSWD group required fewer blood transfusions compared to the control group (Fig. 3c). Among the 82 patients in the THSWD group, 8 experienced delirium, whereas in the control group of 79 patients, 31 experienced delirium (*P* < 0.001) (Fig. 3d). However, there was no statistically significant difference in the onset time of delirium between the two groups (Fig. 3e). Additionally, the THSWD group had a shorter duration of delirium compared to the control group (Fig. 3f). At three months post-surgery, the THSWD group displayed a higher Harris Hip Score (HHS) compared to the Control group. Although the THSWD group still had a higher HHS at 12 h post-surgery, there was no statistically significant difference (Fig. 3g). The forest diagram in Fig. 3h showed that Age, ADL scores, BMI, duration from admission to surgery, surgical duration, blood loss, transfusion volume, HB, ALB, and BUN did not have statistically significant associations with POD. Conversely, CRP levels exhibited a significant association with POD (HR = 1.318, 95% CI: 1.073–1.619, *p* = 0.009). Furthermore, BCR demonstrated a significant association with POD (HR = 2.612, 95% CI: 1.376–4.956, *p* = 0.003).

### Building a protein-protein interaction network and investigating potential pathways for the prevention and treatment of delirium by THSWD

**Fig. 4:**
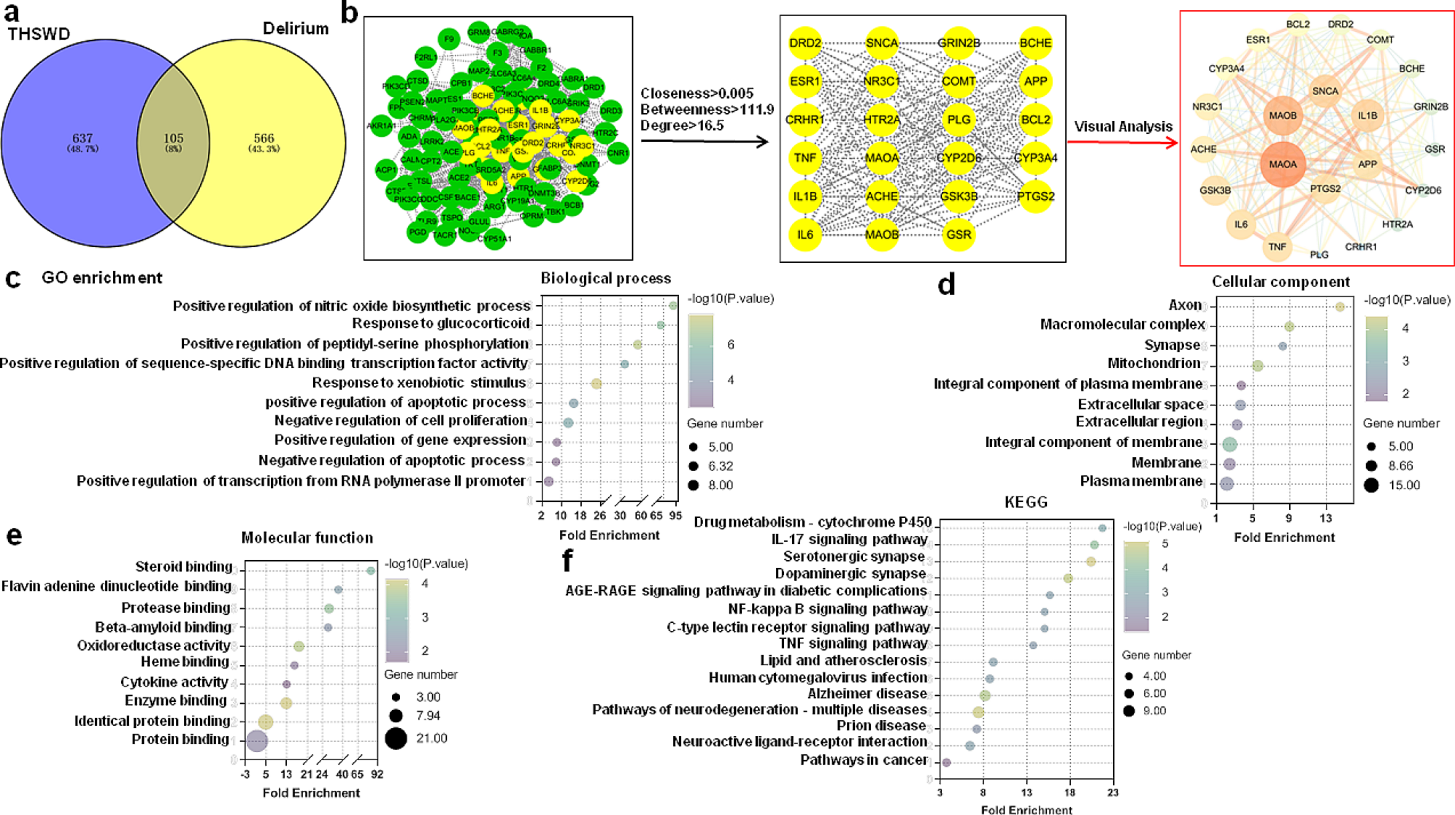
Building a protein-protein interaction network and investigating potential pathways for the prevention and treatment of delirium by THSWD **a** Overlaps between THSWD targets and targets in delirium. **b** PPI network involving THSWD and delirium-related targets. **c-e** GO enrichment analysis. **f** Enrichment analysis of KEGG pathways

A total of 105 potential targets of THSWD associated with delirium were imported into the STRING database to construct a protein-protein interaction (PPI) network model (Fig. 4c-f).

### Target network and molecular docking of THSWD’s active ingredients for delirium

**Fig. 5:**
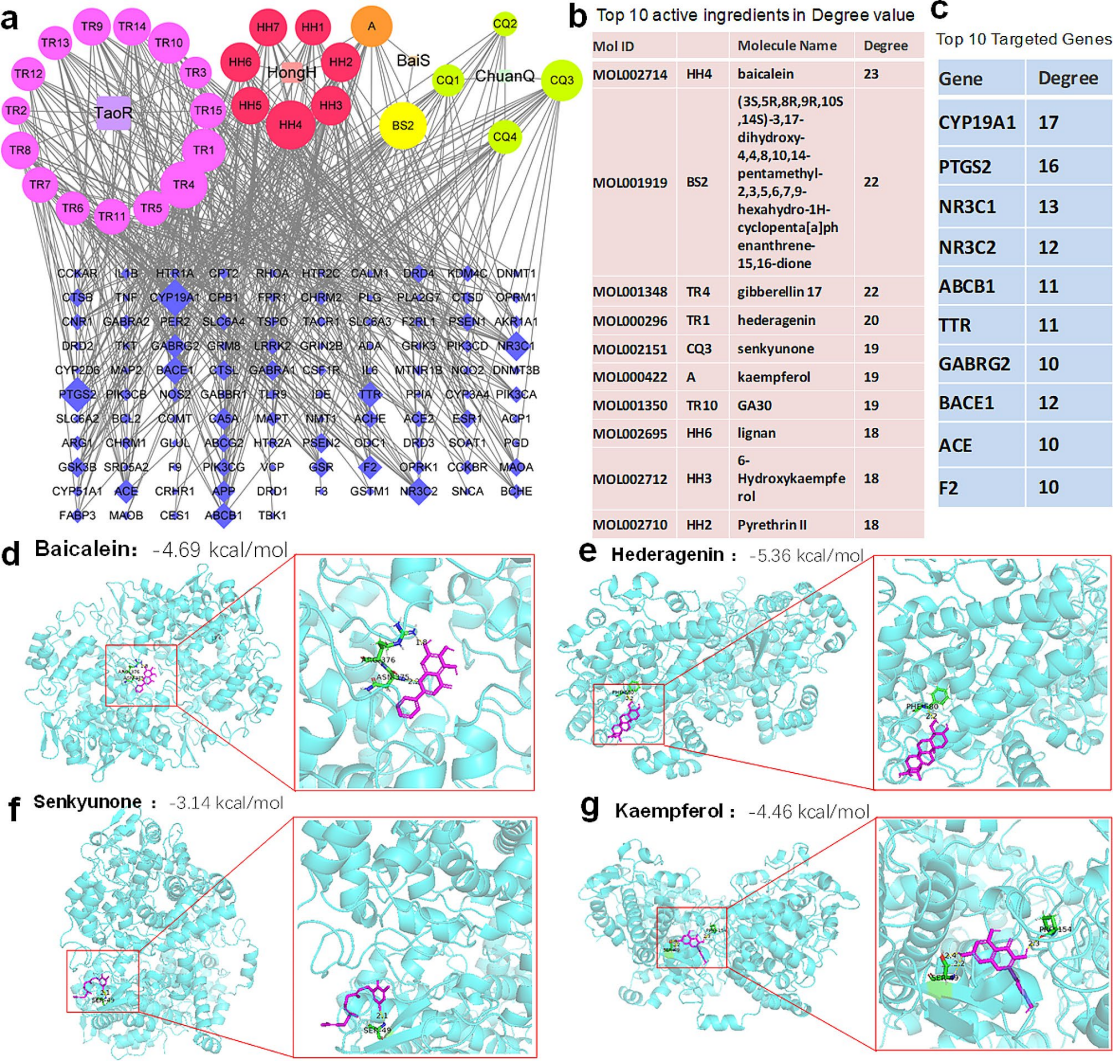
Target network and molecular docking of THSWD’s active ingredients for delirium. **a** Chinese medicine-active ingredient-target network **b** The top ten active ingredients based on their degree value **c** The top ten targets based on their degree value **d-g** Molecular docking revealed the binding of baicalein, hederagenin, senkyunone and kaempferol to the COX-2 protein(blue)

Using the screening criteria of OB ≥ 30% and DL ≥ 0.18%, a total of 30 active ingredients were identified on the SwissADME platform. These included 16 from Taoren (TR1-TR15), 9 from Honghua (HH1-HH7 and A), 2 from Baishao (A and BS2), and 4 from Chuanxiong (CQ1-CQ3). There was 1 shared active ingredient between Honghua and Baishao. Mapping the active ingredient targets with delirium targets resulted in 105 interacting targets. A Chinese medicine-active ingredient-target network was created using Cytoscape 3.8.2 software. The network consisted of 137 nodes and 473 edges (Fig. 5c). PTGS2 is the gene that encodes cyclooxygenase-2 (COX-2). The active ingredients (MOL002714, MOL000296, MOL002151, MOL000422) undergo molecular docking with COX-2. The molecular docking results for COX-2 (blue) and baicalein (red) indicate the formation of hydrogen bonds at COX-2 ASN375 and ARG376, with affinity values of -4.69 kcal/mol(Fig. 5d). Additionally, Fig. 5e and f demonstrate the interaction of the active ingredients hederagenin and senkyunone with the hydrogen bonding sites PHE580 and SER49 of COX-2, exhibiting affinity values of -5.36 kcal/mol and − 3.14 kcal/mol, respectively. Furthermore, kaempferol forms hydrogen bond sites with COX-2 at SER49 and PRC154, exhibiting an affinity value of -4.46 kcal/mol(Fig. 5g). The above molecular docking results suggest that the active ingredients in THSWD exhibit strong binding affinity with COX-2.

### THSWD could downregulate the expression of inflammatory factors in the blood of patients with POD

**Fig. 6:**
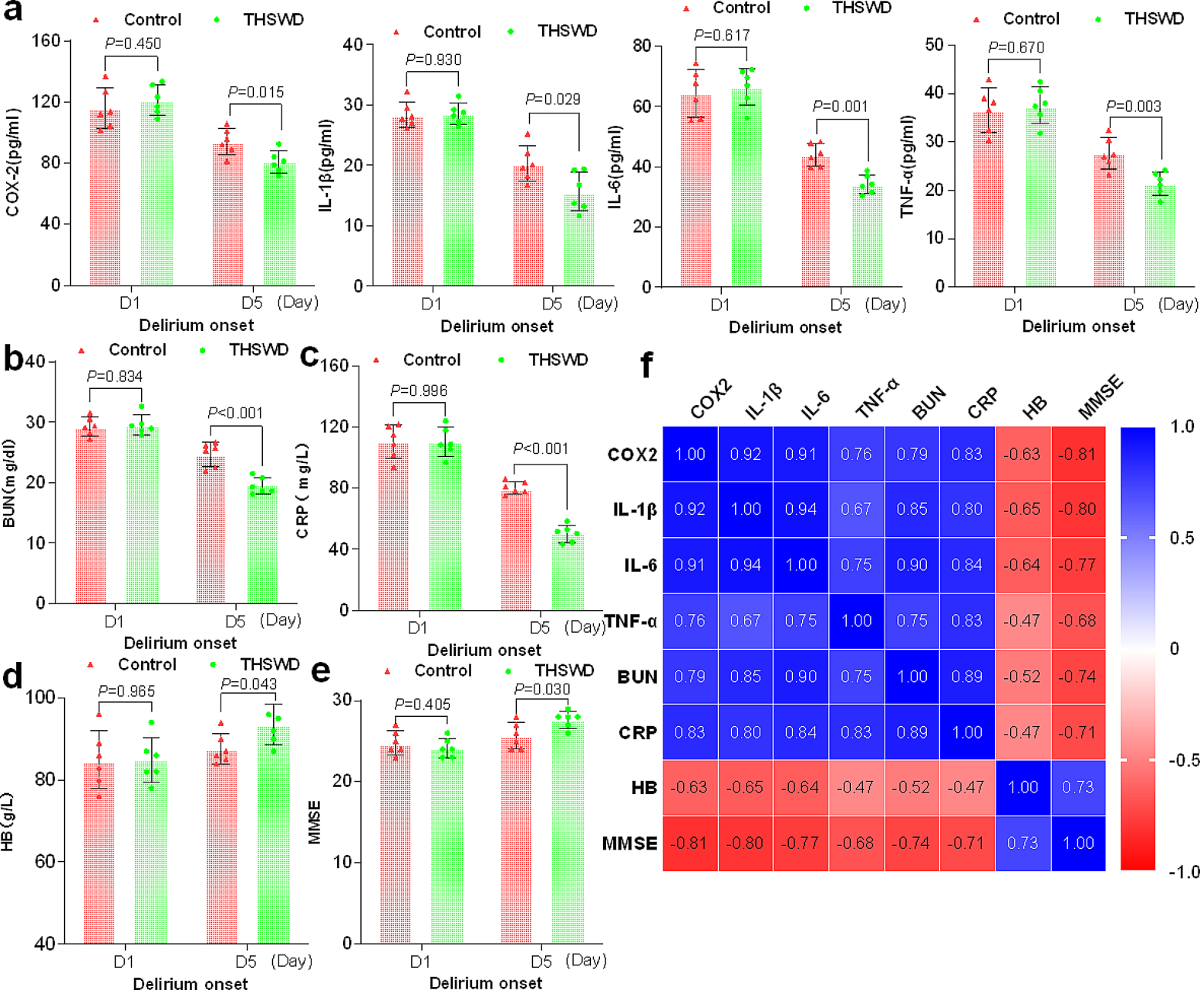
THSWD could downregulate the expression of inflammatory factors in the blood of patients with POD **a** The levels of COX-2 IL-1β, IL-6, and TNF-α were detected using ELISA on both the day of delirium onset (D1) and the 5th day after delirium onset. *n* = 6.**b-d** Expression levels of BUN, CRP, and HB in the blood were measured. **e** MMSE (Mini-Mental State Examination) scores **f** Heat map illustrating the correlation among different indicators

The results mentioned above suggest that THSWD can help reduce the occurrence of POD and shorten its duration. Furthermore, network pharmacology indicates that THSWD may prevent POD by regulating inflammatory factors such as IL1B, PTGS2, TNF, and IL6. To further investigate the therapeutic effect of THSWD on POD, we administered THSWD treatment on the day of delirium onset. We measured the expression of COX-2, IL-1β, IL-6, and TNF-α in the blood using ELISA on both the day of delirium onset (D1) and the 5th day after delirium onset (D5). There were no significant differences in gender, age, ADL scores, BMI, duration from admission to surgery, Surgical duration and Blood loss between the Control group and the THSWD group (Table 2).The results showed that there was no significant difference in the expression of these inflammatory factors (COX-2 IL-1β, IL-6 and TNF-α) between the two groups on the day of delirium onset (before treatment). However, after 5 days of THSWD treatment, the expression of these inflammatory factors in the blood was reduced (Fig. 6f).

**Table 2:** Comparison of general demographics (mean ± SD; *n*, %)

POD	Control (n = 6)	THSWD (n = 6)	*t/*χ2	*P*
Gender			0	1
Male	2(33.33)	2(33.33)		
Female	4(66.67)	4(66.67)		
Age(years)	82.50 ± 2.88	82.17 ± 2.32	0.221	0.830
ADL scores	37.33 ± 2.73	37.17 ± 3.19	0.097	0.925
BMI(kg/cm^2^)	22.14 ± 1.71	22.00 ± 1.76	0.141	0.890
From admission to surgery(days)	3.07 ± 0.58	2.82 ± 0.62	0.720	0.488
Surgical duration	84.17 ± 12.00	82.50 ± 8.80	0.274	0.790
Blood loss	121.67 ± 44.91	141.67 ± 42.15	0.795	0.445

## Discussion

The incidence rates of POD for elderly patients aged 50 and above with hip fractures who undergo spinal anesthesia and general anesthesia are 33.9% and 37.8%, respectively. However, there is no correlation between the type of anesthesia and the functional outcomes [24]. This study findings indicate that the prevalence of POD in elderly patients aged 70 or above, who underwent spinal anesthesia for intertrochanteric fractures, is 39.2%. Advanced age is a significant risk factor for POD, particularly in individuals aged over 80 [25]. Strengthening the prevention of POD is essential because it can lead to longer hospital stays and increased healthcare costs [10–12]. Moreover, around 30–40% of delirium cases can be prevented by implementing several measures, including the identification and management of perioperative risk factors [26], avoidance of specific medications [27], and the provision of comprehensive care [28]. Currently, there is a dearth of effective pharmacological interventions for preventing POD. Although antipsychotic drugs like haloperidol and risperidone have some efficacy, their adverse reactions, including drowsiness, should not be disregarded [6, 13]. Therefore, this study aims to investigate the preventive effect of delirium from the perspective of TCM theory. THSWD promotes blood circulation and removes blood stasis, making it an effective treatment for delirium caused by qi deficiency and blood stasis.

Anemia is strongly associated with delirium, which increases the incidence of delirium with hemoglobin below 97 g/L, whereas correction of anaemia by blood transfusion can reduce the incidence of delirium [29]. Recent research indicates that hip fracture patients under the care of a specialized orthogeriatrician exhibit notably elevated hemoglobin levels upon discharge and have a reduced need for transfusions during hemiarthroplasty [30]. In our results, the THSWD group exhibits elevated hemoglobin levels and requires fewer blood transfusions compared to the control group. These findings indicate that THSWD may contribute to the reduction of bleeding or the promotion of hemoglobin regeneration. In the investigation of abnormal uterine bleeding, it was discovered that THSWD has the ability to decrease the volume of uterine bleeding [31]. Our findings indicate that there is no significant difference in albumin levels between the THSWD group and the control group, despite previous literature suggesting albumin as a risk factor for POD [26]. Furthermore, elevated BUN is a significant risk factor for delirium, with a BCR greater than 24.9 being linked to a higher incidence of delirium [32]. The BUN levels in the THSWD group did not show a significant difference compared to the control group on the day before surgery. However, they were lower on the third day after surgery. The BCR of the THSWD group was lower both on the day before surgery and on the third day after surgery. Additionally, the research has shown that hemoglobin and BUN are the primary risk factors for delirium after microvascular decompression [33]. Current studies indicate a strong correlation between postoperative delirium and C-reactive protein (CRP), a protein that is produced in response to inflammation and tissue damage [34, 35]. Our findings indicate that THSWD is effective in reducing CRP levels on the third day after surgery, compared to the control group. The study on the treatment of angina revealed that THSWD effectively reduced the levels of high-sensitivity C-reactive protein (hs-CRP), triglycerides (TG), total cholesterol (TC), and low-density lipoprotein cholesterol (LDL-C) in serum, when compared to the group that did not use THSWD [36]. Our analysis of risk factors for POD revealed an association between POD and CRP and BCR. THSWD has the potential to improve blood circulation, alleviate congestion, and stimulate the production of new blood cells [37]. This can lead to an increase in hemoglobin levels and a decrease in BUN and CRP, ultimately helping to prevent POD. Moreover, THSWD has the ability to increase the proliferation and osteogenic differentiation of human bone marrow mesenchymal stem cells by activating the VEGF-FAK signaling pathway, thus facilitating the healing process of fractures [38]. In our study, the THSWD group exhibited a higher Harris Hip Score at 3 months post-surgery compared to the Control group.

The exact pathogenesis of delirium remains uncertain, but the prevailing theory suggests that it is associated with neuroinflammation and an imbalance of neurotransmitters [13]. According to TCM, delirium is believed to be caused by qi deficiency and blood stasis. This is because qi deficiency and blood stasis can result in inadequate nourishment of the brain collaterals and blockage of the clear orifices, ultimately leading to delirium episodes. THSWD exhibits neuroprotective effects by potentially reducing the release of inflammatory factors, inhibiting complement signal activation [19], enhancing mitochondrial autophagy, and reducing NLRP3 inflammasome activation [20]. Our research revealed that THSWD, a traditional herbal formula known for its ability to improve blood circulation and resolve stasis, has the potential to prevent POD and reduce its duration. THSWD has the ability to decrease the expression of inflammatory factors, including IL-1β, IL-6, and TNF-α, and also inhibit the activation of inflammasomes [39]. This study discovered that treatment with THSWD can decrease the levels of COX-2, IL-1β, IL-6, and TNF-α in the blood of patients experiencing POD. Network pharmacology analysis identified the top ten target genes for THSWD treatment of delirium, including MAOA, MAOB, SNCA, IL1B, APP, PTGS2, TNF, IL6, GSK3B and ABHE. KEGG enrichment analysis indicates that THSWD has the potential to prevent and treat POD possibly through inflammatory pathways such as IL-17 signaling pathways and NF-kappa B signaling pathways. Moreover, Molecular docking demonstrates that multiple active ingredients of THSWD exhibit strong binding activity with COX-2. COX-2 is a protein that participates in the synthesis of prostaglandins and is involved in physiological processes including inflammation and pain regulation [40]. Our findings suggest a positive correlation between COX-2 and other inflammatory markers (IL-1β, IL-6, TNF-α, and CRP), as well as a negative correlation with MMSE. The presence of elevated levels of inflammatory factors in the brain results in the deterioration of extensive neural networks, including the inhibition of hippocampal plasticity and neurogenesis [41], neurotoxicity, and neuronal apoptosis [42], ultimately leading to the occurrence and development of POD. Therefore, THSWD has the potential to suppress the expression of inflammatory factors, which can contribute to the prevention and treatment of POD.

Despite the potential of THSWD in preventing and treating postoperative delirium, our study has several limitations. Firstly, the relatively small number of participants in our study may limit the generalizability of the results to the larger population. Secondly, the absence of a control group receiving placebo or alternative treatment makes it challenging to determine the specific effect of THSWD and differentiate it from other influencing factors. Finally, the subsequent step of the experiment involves measuring the expression levels of MAOA, MAOB, CYP19A1, SNCA, etc.

## Conclusions

THSWD administration may reduce the incidence and duration of POD by potentially increasing hemoglobin levels, decreasing blood urea nitrogen, and mitigating inflammatory factors. Additionally, network pharmacology analysis indicates that THSWD may offer preventive and therapeutic approaches for POD by modulating inflammatory factors including COX-2, IL-1β, IL-6, and TNF-α.

## Data Availability

No datasets were generated or analysed during the current study.
